# Shape-dependent cellular toxicity on renal epithelial cells and stone risk of calcium oxalate dihydrate crystals

**DOI:** 10.1038/s41598-017-07598-7

**Published:** 2017-08-03

**Authors:** Xin-Yuan Sun, Jian-Ming Ouyang, Kai Yu

**Affiliations:** 1 Department of Chemistry, Jinan University, Guangzhou, 510632 China; 2 Institute of Biomineralization and Lithiasis Research, Jinan University, Guangzhou, 510632 China

## Abstract

Renal epithelial cell injury causes crystal retention and leads to renal stone formation. However, the effects of crystal shape on cell injury and stone risk remain unclear. This study compared the cytotoxicity degrees of calcium oxalate dihydrate (COD) crystals having different shapes toward human kidney proximal tubular epithelial (HK-2) cells to reveal the effect of crystal shape on cell injury and to elucidate the pathological mechanism of calcium oxalate kidney stones. The effects of exposure to cross-shaped (COD-CS), flower-like (COD-FL), bipyramid (COD-BD), and elongated–bipyramid (COD-EBD) COD crystals on HK-2 cells were investigated by examining the cell viability, cell membrane integrity, cell morphology change, intracellular reactive oxygen species, mitochondrial membrane potential (Δψm), and apoptotic and/or necrotic rate. Crystals with large (100) faces (COD-EBD) and sharp edges (COD-CS) showed higher toxicity than COD-BD and COD-FL, respectively. COD crystal exposure caused cell membrane rupture, upregulated intracellular reactive oxygen, and decreased Δψm. This series of phenomena ultimately led to a high apoptotic rate and a low necrotic rate. Crystals with large active faces have a large contact area with epithelial cell surface, and crystals with sharp edges can easily scratch epithelial cells; these factors could promote crystal adhesion and aggregation, thus increasing stone risk.

## Introduction

Kidney stone formation is a complex biological regulation process that usually includes crystal nucleation, growth, aggregation, and retention^1^. More than 80% of kidney stones are calcium oxalate (CaOx) stones in the form of calcium oxalate monohydrate (COM) and calcium oxalate dihydrate (COD). COD is the second most popular type of kidney stone and the most frequent CaOx crystal present in the urine of patients with idiopathic calcium urolithiasis^2^.

Kidney stones often differ in shape, size, and crystal phases depending on the degree of urinary supersaturation, concentrations of inhibitors and enhancers, and retention time of microcrystals^3–5^. In recurrent stone formers, CaOx crystallites mainly comprise aggregated octahedral COD crystals 10–12 μm in size with sharp edges. In non-stone formers, CaOx is mainly in the form of small blunt crystals 3–4 μm in size with few aggregation^3^. In addition, crystallites are mostly dispersed and spheroid in healthy urine samples but feature sharply angled edges and tips in lithogenic urine samples due to the lack of urinary inhibitors^5^.

Recent studies have demonstrated that the cytotoxicity of CaOx crystals toward renal epithelial cells is closely related to crystal phase and size^6, 7^. COM crystals cause more serious injury to renal epithelial cells than same-sized COD crystals^6^. Furthermore, the cytotoxic effect of COD crystals on renal epithelial cells is size dependent and exacerbates in the following order: 50 nm > 100 nm > 600 nm > 3 μm > 10 μm^7^. Small crystallites are easier to aggregate than large crystallites, and aggregates with small primary sizes are larger than those with large primary sizes^8^. Particle shape, which is a considerable physical parameter for crystals, may also play an important effect on the interaction between micro-/nanosized particles and cells. To date, the effects of CaOx crystal shape on their cytotoxicity and the risk of inducing stone formation remain unclear.

The shape of exogenous particles is an important parameter influencing their biological safety and application^9–12^. For instance, a study conducted on zebrafish embryos found that 30, 60, and 100 nm spherical nickel nanoparticles are less toxic than 60 nm dendritic clusters. This study suggests that the configuration of nanoparticles affects their toxicity more than size, and defects due to nanoparticle exposure occur through different biological mechanisms^10^. Zhang *et al*. compared the toxicities of 3D spherical and 2D discoid polystyrene nanoparticles^11^. Compared with nanospheres, nanodisk particles promote cell surface binding with significant reduction of cell uptake, thus eliciting minimal perturbations on cell functions, such as cellular reactive oxygen species (ROS) generation, apoptosis, and cell cycle progression. Particle shape could also affect cell uptake. Huang *et al*. reported that mesoporous silica nanoparticles with high aspect ratios are taken up in large amounts and demonstrate fast internalization rates^12^. They also found that particles with high aspect ratios exert significant effects on cellular processes, including proliferation, apoptosis, cytoskeleton formation, adhesion, and migration.

The toxicity differences of particles with varying shapes may be affected by adhesion strength to cells, internalization rate, and margination dynamics in the blood vessels^13^. Controlling material morphology is important to elucidate the pathological mechanisms of diseases caused by exogenous and endogenous particles, such as kidney stone, gall stone, and atherosclerosis. However, limited studies have reported on the geometry-dependent toxicity of particles and the mechanism of pathogenic mineralization diseases. Accordingly, we compared the cytotoxicity degrees of COD crystals with varying shapes (cross-shaped, flower-like, bipyramid, and elongated–bipyramid) toward human kidney proximal tubular epithelial (HK-2) cells to reveal the effect of COD crystal shape on kidney stone formation.

## Results

### Preparation and characterization of COD crystals with different shapes

**Figure 1:**
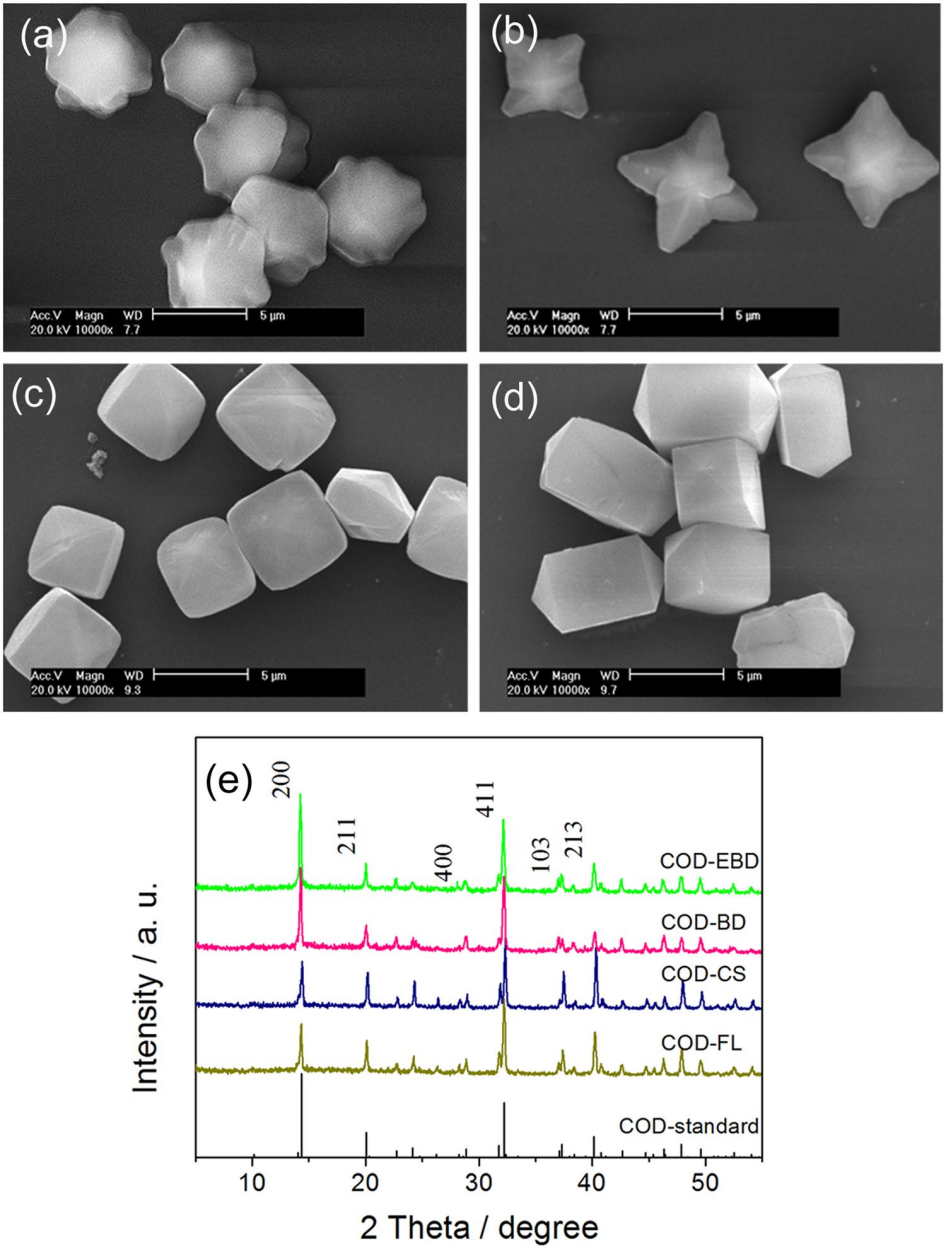
SEM images (**a**–**d**) of COD crystals with various shapes and the XRD patterns (**e**) of as-prepared COD crystals and the standard XRD pattern of COD (PDF card number: 17-0541)^14^. (**a**) COD-FL; (**b**) COD-CS; (**c**) COD-BD; (**d**) COD-EBD; (**e**) XRD spectra. Scale bars: 5 μm.

COD crystals with different shapes were prepared by altering reactant concentration, reaction temperature, stirring speed, and additive. Table 1 shows the additives during the preparation process and physicochemical characteristics of COD crystals. Figure 1a–d illustrate the SEM images of the COD crystals (~5 µm in size) with different shapes, including cross-shaped (COD-CS), flower-like (COD-FL), bipyramid (COD-BD), and elongated–bipyramid (COD-EBD). Both COD-CS and COD-FL presented a similar dendrite structure, of which COD-CS comprised four sharp branches, whereas COD-FL had eight blunt branches. COD-BD and COD-EBD crystals showed the most common morphology of bipyramid structure, but the (100) face was gradually extended. Figure 1e shows the XRD patterns of the as-prepared COD crystals of varying shapes and the standard XRD pattern (PDF card number: 17-0541)^14^. All crystals were detected at diffraction peaks d = 0.618, 0.442, 0.277, 0.241, and 0.224 nm, which were assigned to the (200), (211), (411), (103), and (213) planes of COD crystals, respectively. No other impurity peaks in the XRD spectra were found. The intensity ratio of the main (100) and (211) planes (I_100_/I_211_) of the COD crystals with varying shapes was COD-EBD > COD-BD > COD-FL > COD-CS (Table 1).

**Table 1:** Characterization of the physical and chemical properties of COD crystals with various shapes.

Crystal morphology and abbreviation	Crystal size/μm	Additive	I_100_/I_211_	Specific surface area S_*BET*_ /m^2^/g	Zeta potential in culture medium/mV	Conductivity/μS/cm
COD-flower-like (COD-FL)	4.8 ± 0.3	Na_2_EDTA	1.46	2.79	−6.01 ± 1.32	43.3 ± 0.92
COD-cross-shaped (COD-CS)	5.2 ± 0.5	Na_2_EDTA	1.33	3.04	−5.75 ± 1.19	48.2 ± 1.17
COD-bipyramid (COD-BD)	4.9 ± 0.2	SDS	3.43	5.33	−12.6 ± 1.68	35.7 ± 0.74
COD-elongated-bipyramid (COD-EBD)	4.7 ± 0.4	SDS	4.10	5.81	−10.9 ± 2.62	33.6 ± 1.85

Table 1 also shows the specific surface area (S_*BET*_), conductivity, and zeta potentials of the crystals in culture medium. Lesser physical property differences were observed in the crystals obtained by the same additive than in the crystals obtained by different additives. COD-EBD had slightly higher S_*BET*_ (5.81 *vs* 5.33) and slightly lower absolute values of zeta potentials (10.9 *vs* 12.6) than COD-BD. Meanwhile, COD-CS had slightly higher S_*BET*_ (3.04 *vs* 2.79) and slightly lower absolute values of zeta potentials (5.75 *vs* 6.01) than COD-FL. In this study, we mainly discuss the toxicity difference of the crystals obtained by the same additive because the physical property difference was relatively small and because the different additive absorptions during crystal preparation may affect their toxicity.

### Cell viability changes caused by COD crystals with various shapes

**Figure 2:**
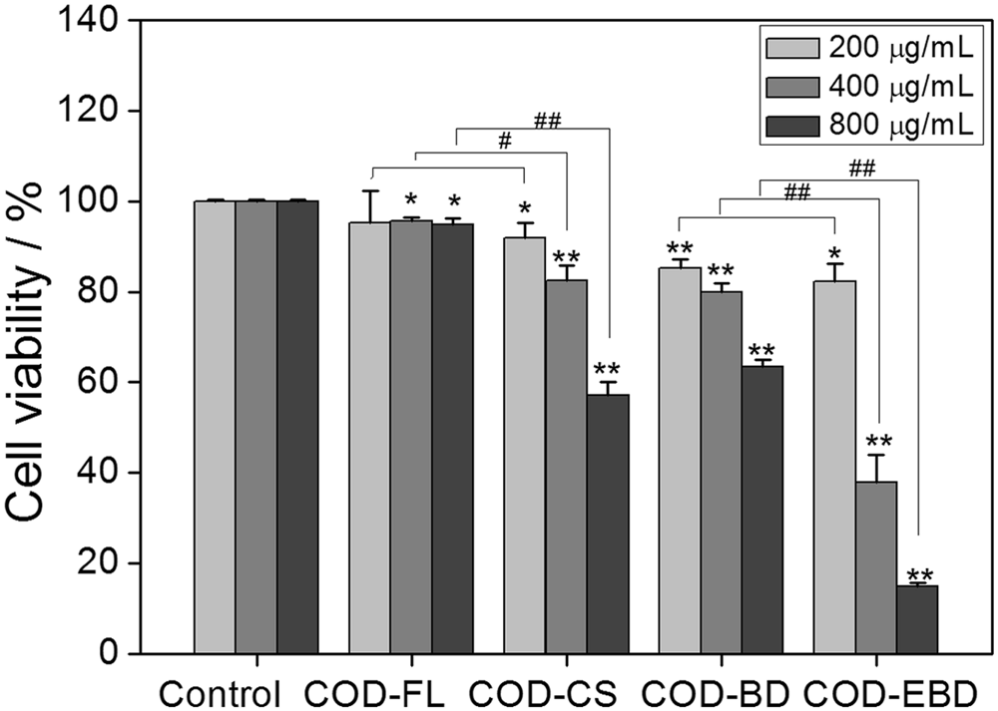
Cell viability detection by CCK-8 assay of HK-2 cells after exposure to different concentrations of COD with various shapes for 6 h. Compared with control group, *p < 0.05. **p < 0.01. COD-FL treatment group *vs* corresponding concentration of COD-CS treatment group, COD-BD treatment group *vs* corresponding concentration of COD-EBD treatment group, ^#^P < 0.05, ^##^P < 0.01.

To compare the cytotoxicity of COD crystals with various shapes in renal epithelial cells, we used CCK-8 assay to detect cell viability (Fig. 2). The adopted concentration of the crystals ranged from 200 μg/mL to 800 μg/mL, which was consistent with previous study^15^. The COD-CS and COD-FL crystals at a low concentration of 200 μg/mL showed slight differences in cytotoxicity. The cytotoxicity of COD-CS increased rapidly with increasing crystal concentration, but the cytotoxicity changes in the COD-FL-treated group were not obvious. The toxicity of COD-CS was significantly higher than that of COD-FL when the crystal concentration was increased to 400 μg/mL (*p* < 0.05) and 800 μg/mL (*p* < 0.01). Furthermore, the cell viability of the COD-EBD-treated group decreased more rapidly than that of the COD-BD-treated group when the crystal concentration was increased. The toxicity of COD-EBD in HK-2 cells was significantly higher than that of COD-BD at high concentrations (*p* < 0.01).

### Cell morphology changes caused by COD crystals with various shapes

**Figure 3:**
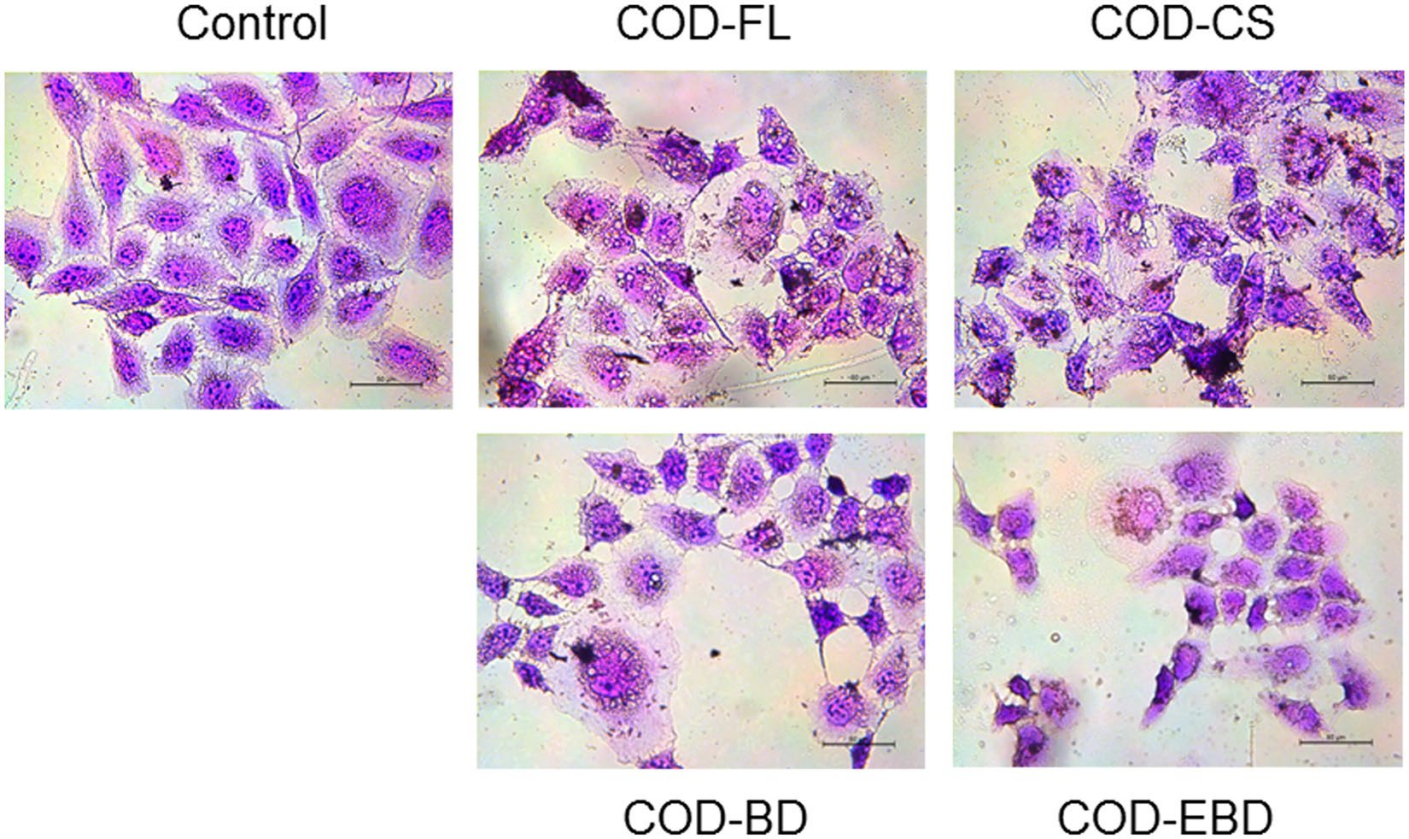
Morphology observation by HE staining of HK-2 cells after exposure to 400 μg/mL COD crystals with various shapes for 6 hours. Scale bars: 50 μm.

Changes in cell morphology could directly reflect the degree of cell damage. Thus, we observed the overall morphology of normal cells and cells treated with COD crystals through HE staining assay (Fig. 3). The cells in the control group presented a plump spindle shape, and the cytoplasm was stained uniformly. By contrast, the morphology of the cells treated with 400 μg/mL COD crystals in various shapes became disordered and presented chromatin condensation and eosinophilic staining enhancement, accompanied by apoptotic body formation. Among the crystals, the COD-EBD crystals caused the most serious damage to HK-2 cells, causing tight junction fracture and morphological disorder. Crystal adhesion was also observed (Fig. 3). Most of the adhered crystals appeared to be flat on the surface of the cell islands. Schepers *et al*. reported that crystals mainly lay on the surface of the cell islands formed by proximal tubule cells, whereas crystals are predominantly found at the periphery of the cell groups formed by collecting duct cells^16^.

### Lactate dehydrogenase (LDH) release caused by COD crystals in various shapes

**Figure 4:**
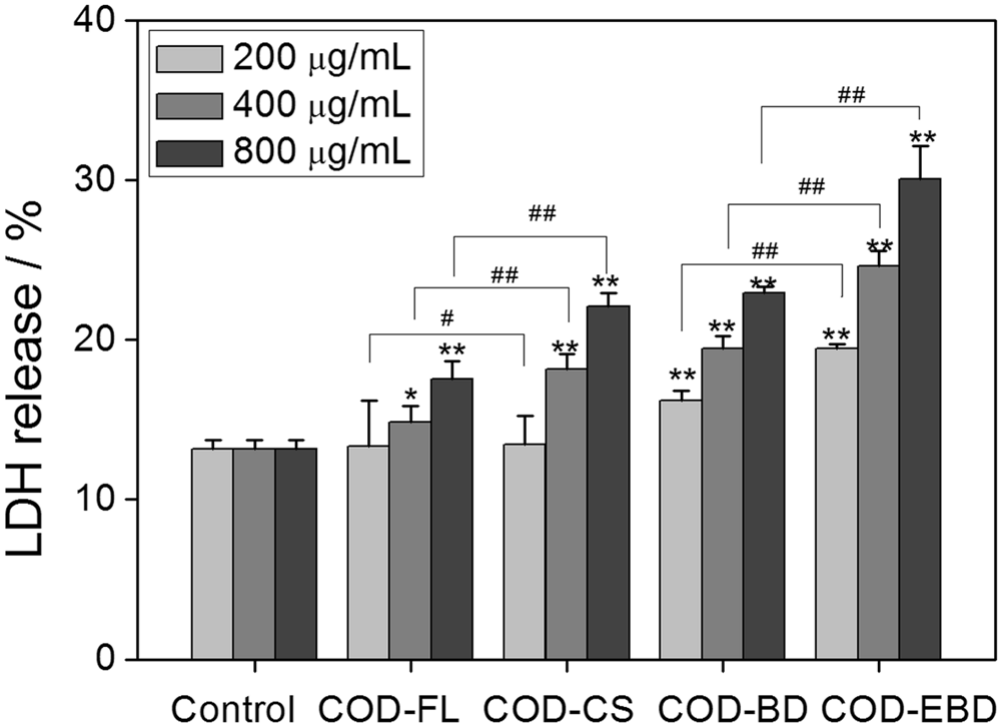
Changes in LDH release amount of HK-2 cells caused by different concentrations of COD crystals with various shapes for 6 h. Compared with control group, *p < 0.05. **p < 0.01. COD-FL treatment group *vs* corresponding concentration of COD-CS treatment group, COD-BD treatment group *vs* corresponding concentration of COD-EBD treatment group, ^#^P < 0.05, ^##^P < 0.01.

Plasma membrane damage is an important aspect of cellular toxicity upon particle treatment. All types of crystals caused the release of intracellular LDH in varying degrees. The released amount increased with increasing crystal concentration (Fig. 4). The COD-CS crystals caused a higher amount of LDH release than the COD-FL crystals, especially when the crystal concentration exceeded 400 μg/mL (*p* < 0.01). In addition, the COD-EBD crystals caused a higher amount of LDH release than the COD-BD crystals at the three concentrations. Among the groups, the COD-EBD-treated group showed the most serious damage to the cell membrane and had the highest amount of LDH release. These results are consistent with the cytotoxicity variation detected using CCK-8 assay.

### Increase in ROS generation induced by COD crystals in various shapes

**Figure 5:**
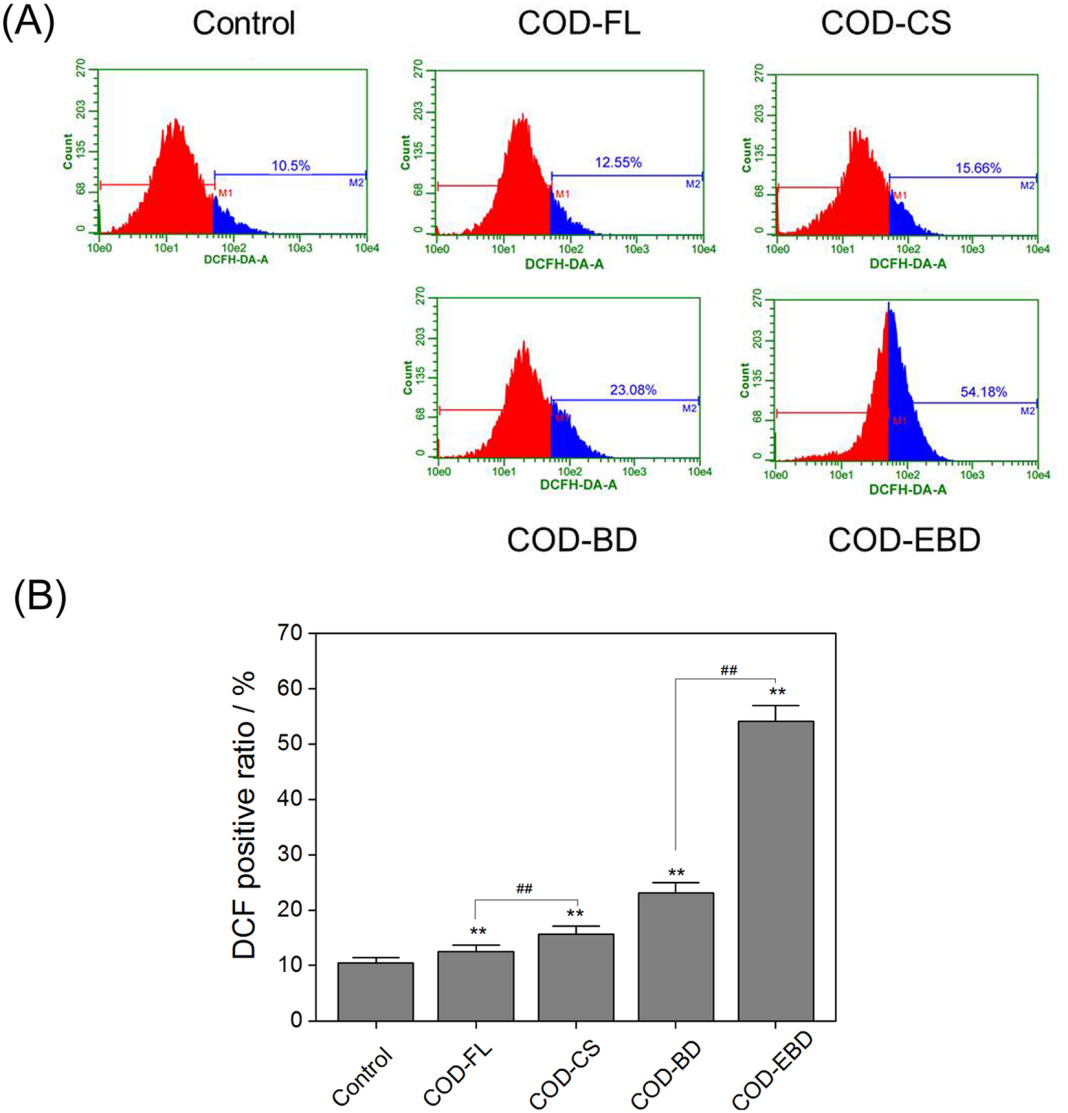
Intracellular ROS level of HK-2 cells after exposure to 400 μg/mL COD crystals with various shapes for 6 hours. (**A**) Histogram of intracellular ROS; (**B**) quantitative results of intracellular ROS. Compared with control group, *p < 0.05. **p < 0.01. COD-FL treatment group *vs* corresponding concentration of COD-CS treatment group, COD-BD treatment group *vs* corresponding concentration of COD-EBD treatment group, ^#^P < 0.05, ^##^P < 0.01.

The amount of intracellular ROS is commonly used to assess particle toxicity. The intracellular ROS in the crystal-treated groups was labeled using DCFH-DA and analyzed via flow cytometry (Fig. 5). Compared with the control group, the four treated groups showed higher ROS levels. The ROS level in the COD-CS-treated group (15.66%) was significantly (*p* < 0.01) higher than that in the COD-FL-treated group (12.55%). The ROS level in the COD-EBD-treated group (54.18%) was also significantly (*p* < 0.01) higher than that in the COD-BD-treated group (23.08%). Among the four treated groups, the COD-EBD-treated group induced the highest ROS generation, and the amount of DCF-positive cells reached 54.18%. The COD-EBD crystals with a large (100) face and the COD-CS crystals with sharp edges induced higher ROS generation than the other crystals.

### Decrease in mitochondrial membrane potential (Δψm) caused by COD crystals in various shapes

**Figure 6:**
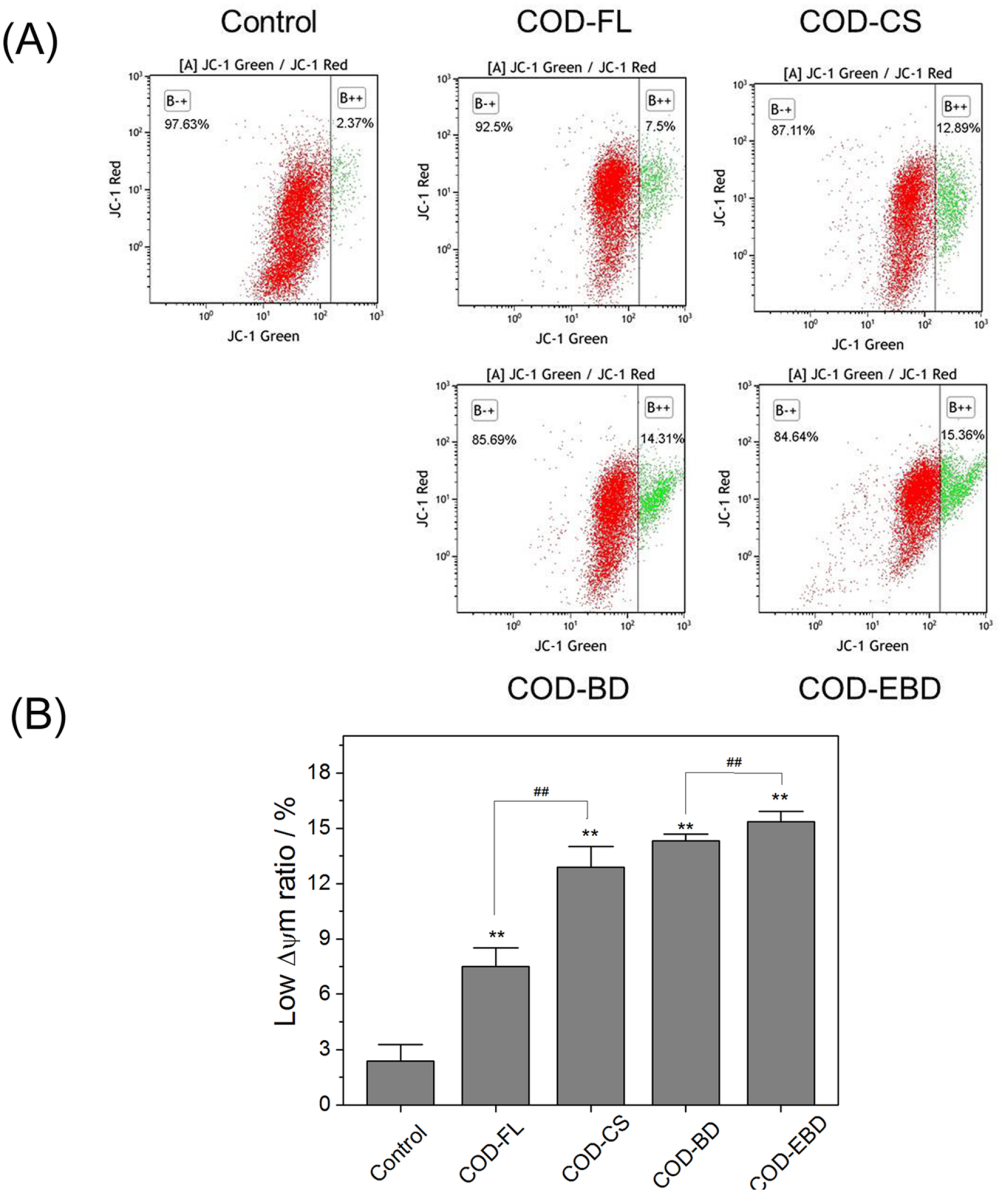
Effect of varying shapes of COD crystals on mitochondrial membrane potential (Δ*ψ*m) in HK-2 cells. (**a**) Dot plot of Δ*ψ*m after incubation with varying shapes of COD crystals for 6 h; (**b**) quantitative histogram of Δ*ψ*m. Crystal concentration: 400 μg/mL. Compared with control group, *p < 0.05. **p < 0.01. COD-FL treatment group *vs* corresponding concentration of COD-CS treatment group, COD-BD treatment group *vs* corresponding concentration of COD-EBD treatment group, ^#^P < 0.05, ^##^P < 0.01.

Mitochondria have high Δψm potential under normal circumstances and become depolarized after suffering injury. Therefore, we analyzed the changes in Δψm in the cells treated with COD crystals of various shapes by JC-1 fluorescent staining and flow cytometry (Fig. 6).

The ratio of cells with low Δψm (green fluorescent) in the control group was only 2.37%, but this value was increased upon treatment with the COD crystals in various shapes. This result indicates that COD crystal exposure caused different degrees of mitochondrial depolarization. The COD-FL-treated group showed a 7.5% decrease in ΔΨm, whereas the COD-CS-treated group exhibited a 12.89% decrease in Δψm. The COD-BD and COD-EBD crystals with large (100) planes caused greater decreases in Δψm. In specific, the COD-EBD-treated group demonstrated the most decrease in Δψm (15.36%). Excessive ROS generation may be the main reason for the decrease in Δψm. The change law of Δψm decrease was similar to that of ROS increase.

### Cell apoptosis and necrosis induced by COD crystals in various shapes

**Figure 7:**
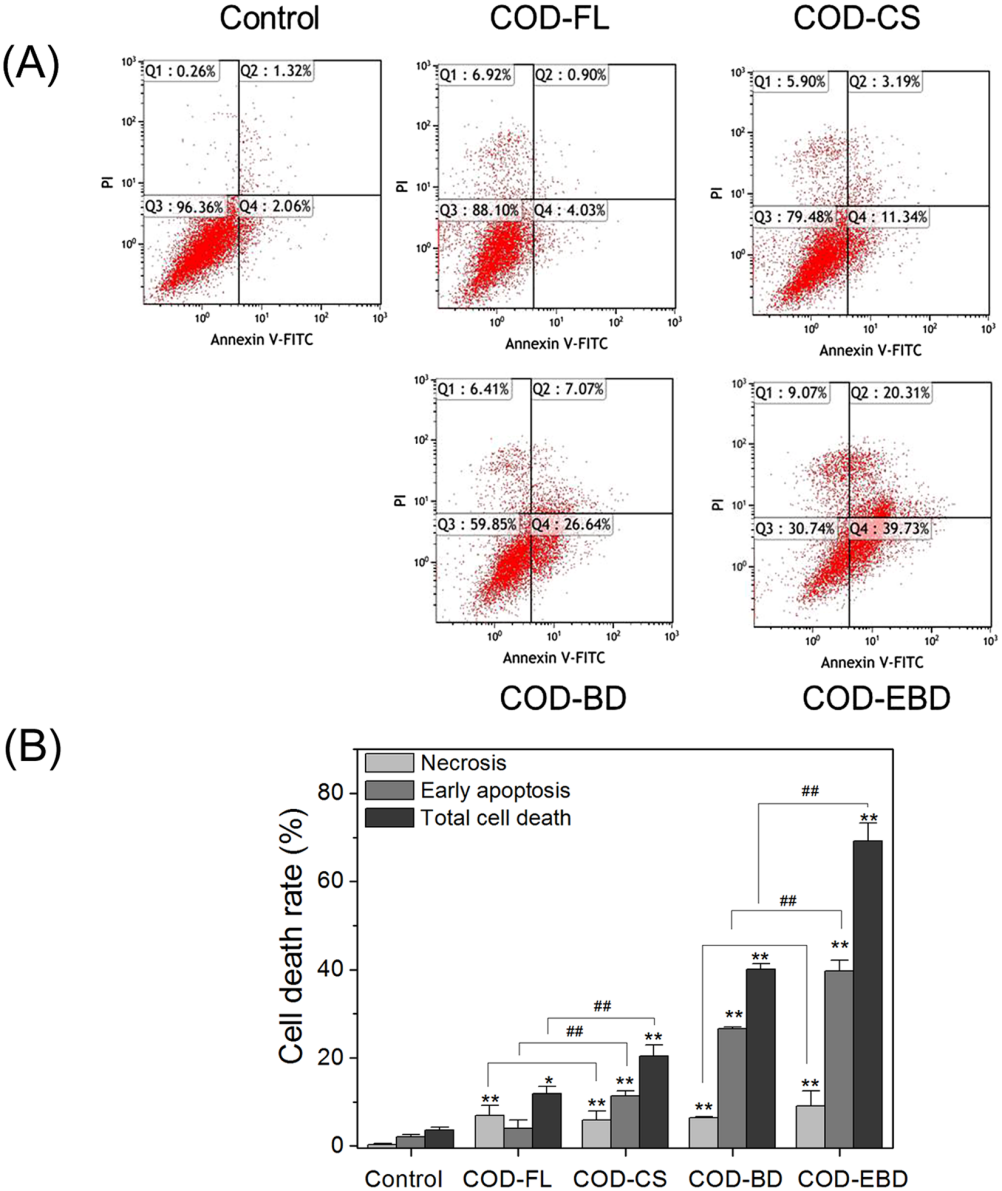
Cell death of HK-2 cells after exposure to varying shapes of COD crystals for 12 h. (**A**) The representative dot plot of apoptosis and necrosis. Quadrant Q1, Q2, Q3 and Q4 denote the ratio of necrotic cells, late apoptotic cells and/or necrotic cells, normal cells, and early-stage apoptotic cells, respectively. (**B**) Quantitative results of apoptosis and necrosis. Crystal concentration: 400 μg/mL. Compared with control group, *p < 0.05. **p < 0.01. COD-FL treatment group *vs* corresponding concentration of COD-CS treatment group, COD-BD treatment group *vs* corresponding concentration of COD-EBD treatment group, ^#^P < 0.05, ^##^P < 0.01.

Apoptotic and necrotic cells were quantified through flow cytometric analysis using Annexin V/PI double staining to assess the nature of cell death induced by COD crystals with various shapes (Fig. 7). The exposure time was extended to 12 h to distinguish the changes in cell apoptotic and necrotic rates. Quadrants Q1, Q2, Q3, and Q4 denote the ratio of necrotic cells, late apoptotic cells and/or necrotic cells, normal cells, and early apoptotic cells, respectively. Compared with the control, the HK-2 cells treated with COD crystals in various shapes exhibited different degrees of apoptosis and necrosis. The early apoptotic rate in the COD-CS-treated groups was 11.34%, which was significantly higher than that in the COD-FL-treated group (4.03%). The COD-BD and COD-EBD crystals with large (100) planes induced higher cell apoptotic rates than the two other types of crystals. The early apoptotic rates in the COD-BD and COD-EBD-treated groups were 26.64% and 39.73%, respectively. The four COD crystal shapes also partly caused cell necrosis, which may be the reason why the large crystals easily caused cell membrane rupture. Necrotic cells and late apoptotic cells cannot be exactly distinguished by Annexin V/PI double staining only. Quadrant 2 represents the number of PI-positive (necrosis) and Annexin V-positive (apoptosis) cells^17, 18^. Therefore, the rate of necrosis (Quadrant 1) caused by COD crystals is difficult to compare. Statistical analysis results showed that the rate of necrosis was not statistically different between COD-FL and COD-CS and between COD-BD and COD-EBD.

### Determination of crystal adhesion on the cell surface

**Figure 8:**
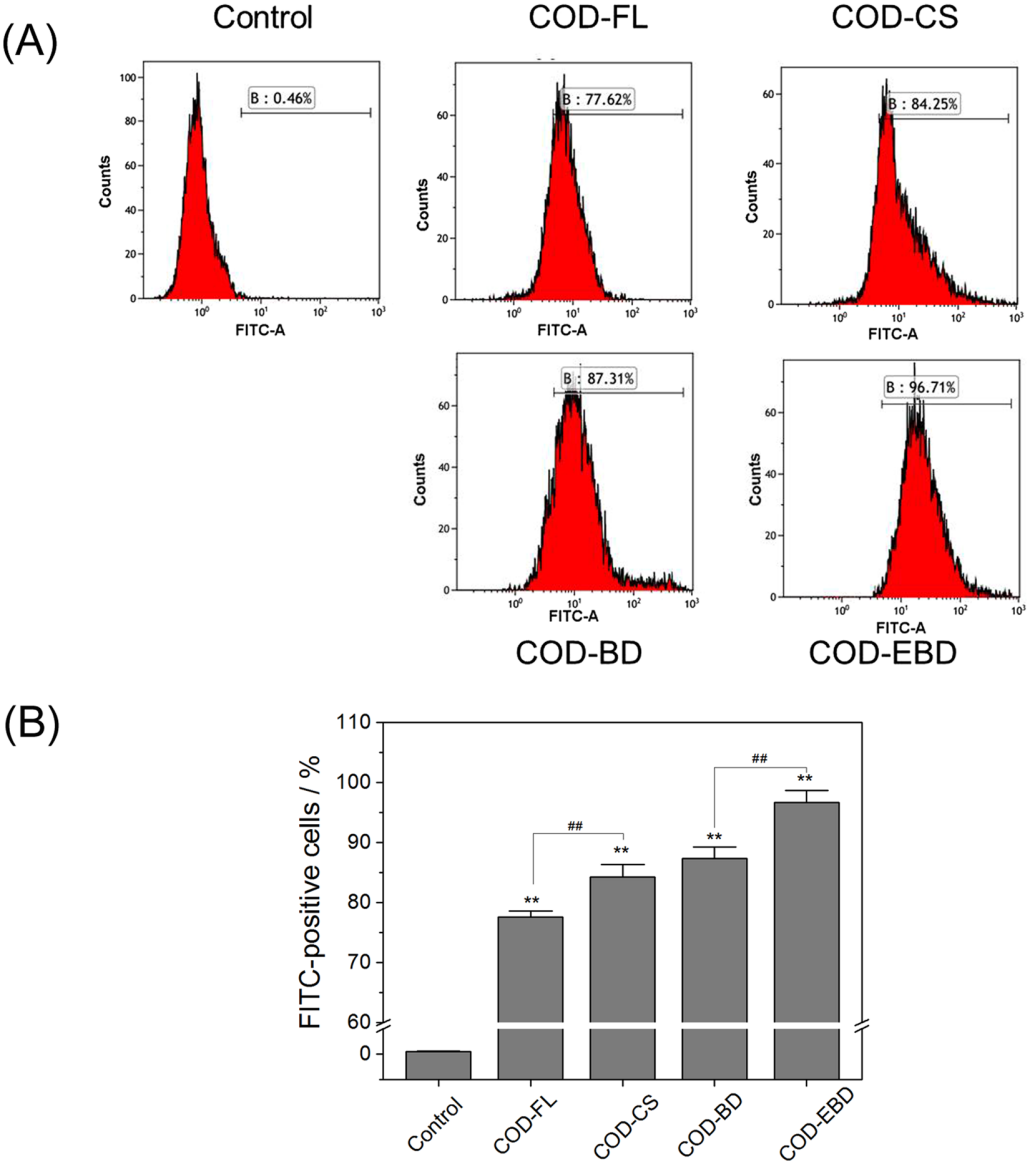
Quantification of adhesion amount of COD crystals with various shapes on cell surface by flow cytometer. (**A**) Histogram of the percentage of FITC-positive cells. (**B**) Quantitative results of adherent crystals. [A] FITC-A means fluorescent intensity; B means percentages of cells with adherent fluorescent COD crystals. Crystal concentration: 400 μg/mL, treatment time: 1 h. Compared with control group, *p < 0.05. **p < 0.01. COD-FL treatment group *vs* corresponding concentration of COD-CS treatment group, COD-BD treatment group *vs* corresponding concentration of COD-EBD treatment group, ^#^P < 0.05, ^##^P < 0.01.

Crystal adhesion on the cell surface is an important stage in stone formation. COD crystals were fluorescently labeled through FITC-IgG, and the number of FITC-positive cells was counted using a flow cytometer (Fig. 8). A crystal adhesion experiment was carried out at 4 °C. Cellular active transport was inhibited under this temperature, and only adhesion proceeded. The adhesion amount of the four types of COD crystals on the HK-2 cell surface was COD-EBD > COD-BD > COD-CS > COD-FL. The adhesion amount is closely related to the crystals’ specific surface area. Crystals with a large specific surface area had a higher adhesion amount on the cell surface compared with those with a small specific surface area.

## Discussion

Urinary crystals in normal individuals and in patients with kidney stones often differ in shape, size, and crystal phases because of differences in physiological environment^3–5^. Aside from the effects of crystal size and phase on cell injury^6, 7^, crystal shape also exerts different effects on cell injury. Previous studies about the effect of crystal shape on cytotoxicity mainly focused on spherical or rod-shaped particles^9, 19^. Crystal planes do not differ in spherical particles, but most crystals formed *in vivo* are polyhedron structures. The crystal planes of nonspherical crystals significantly differ in atomic arrangement and charge distribution. These differences inevitably affect their cell response because of crystal–cell interactions. The urine of renal stone patients usually lacks mineralization inhibitors, which cause crystals with sharp edges to form easily. Meanwhile, the formed crystals in normal subjects are mostly small round particles. Therefore, we prepared COD crystals with different shapes and compared their cytotoxicity degrees through a series of toxicology experiments.

### Cytotoxicity differences of COD crystals in various shapes

Under the regulation of SDS on crystal shape, COD-EBD (2:1) and COD-BD (1:1) with different aspect ratios were obtained. COD-EBD exhibited a larger (100) face than COD-BD. The (100) face enlargement increased the crystal length along the [001] direction. However, large crystals did not present lower toxicity than small crystals, which contradicted the results of a previous study^20^. The cytotoxicity of COD-EBD was obviously higher than that of COD-BD (Fig. 2), indicating that crystal shape contributes more than crystal size to their cytotoxicity^11^. COD-CS and COD-FL crystals with different sharpness were prepared by adding Na_2_EDTA. COD-CS with sharp edges and corners showed higher cytotoxicity than COD-FL.

The cells in the four treated groups exhibited abnormal morphologies (Fig. 3). Typical features of apoptosis, including cellular shrinkage, cell connection disappearance, and nuclear condensation, were observed, especially in the COD-EBD-treated group. The integrity of cell morphology is essential for normal cellular physiological function. In consideration that the plasma membrane is directly linked to and functionally integrated with the underlying actin-based cytoskeleton, cell–crystal interactions that would cause actin rearrangement may be anticipated^12^. The adhesion and endocytosis of COD crystals result in cell membrane disruption and cell cytoskeleton disorganization. The four types of COD crystals all caused LDH release (Fig. 4) to the extracellular space, indicating cell membrane rupture. COD crystals with a large (100) crystal face (COD-EBD) and sharp edges (COD-CS) likely cause cell membrane disruption and intracellular LDH release. The COD-EBD crystals had the largest active (100) face among the four crystals. This characteristic increased the contact area between the crystals and cell surface and directly aggravated exterior damage to the cell membrane. Meanwhile, COD-EBD, which has a large Ca^2+^ ion-rich (100) face, induced substantial ROS generation (Fig. 5), exacerbating interior damage and causing membrane damage. Thus, the COD-EBD-treated group showed the most significant damage to the cell membrane.

**Figure 9:**
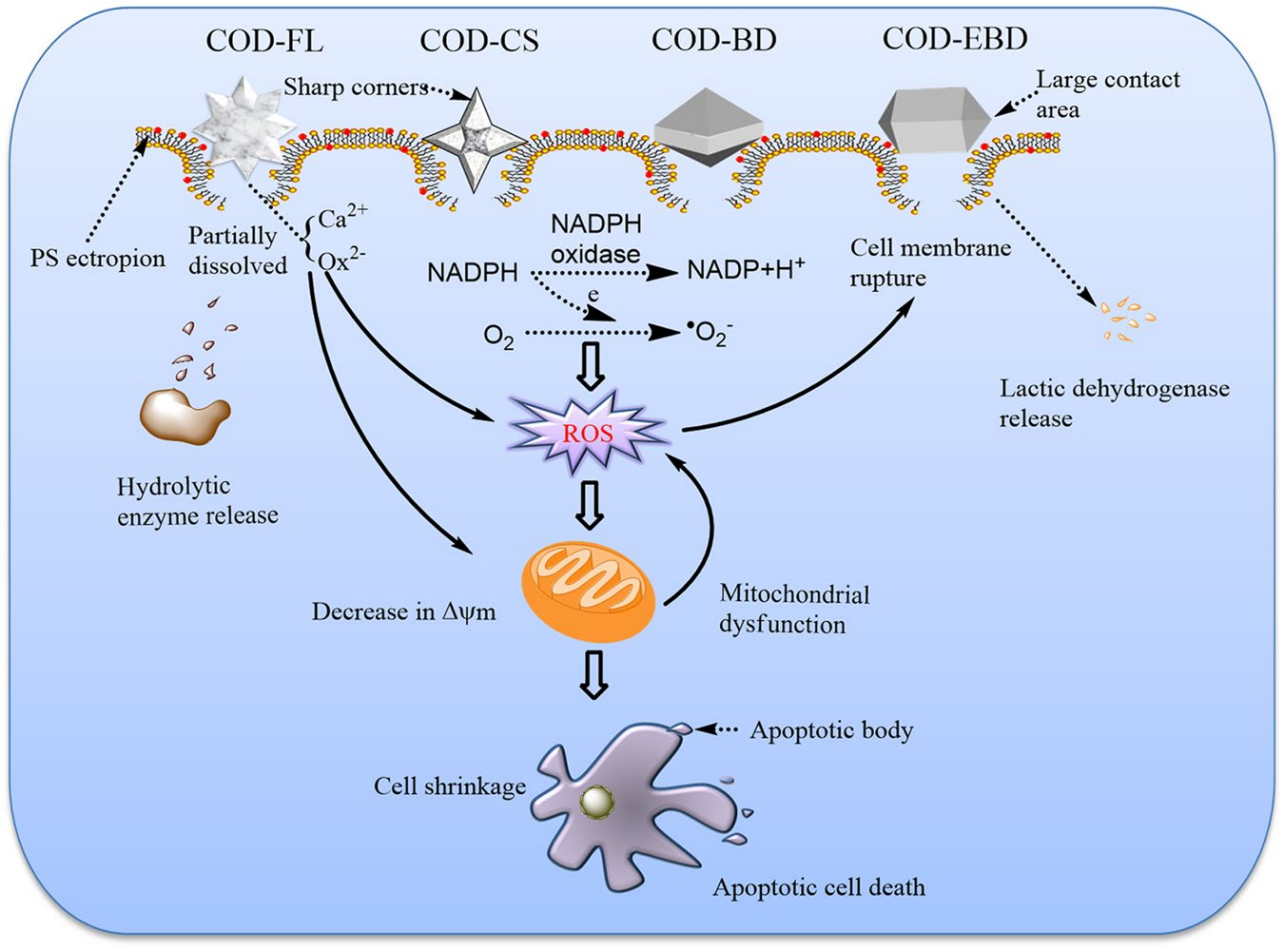
A proposed schematic illustration of the cellular and molecular mechanism of HK-2 cell injury after exposure to varying shapes of COD crystals. Crystals with greater (100) active crystal faces and sharp edges has higher cytotoxicity on renal epithelial cells.

Whether or not COD-induced cell death occurs because of the effect of COD on the membrane or inside the cell has not been established. Therefore, we further detected intracellular biological indicators to reveal the injury mechanism of the different COD crystal shapes. Figure 9 summarizes the proposed schematic of the injury mechanism. In general, ROS generation and oxidative stress produced by exogenous particles are important factors associated with particle toxicity^20^. A direct relationship exists between surface area and ROS generation capability. A larger surface area per unit mass leads to a greater number of atoms or molecules to be displayed on the surface instead of the interior of particles. Therefore, compared with COD-BD and COD-FL, COD-EBD and COD-CS exposed more active sites on the crystal surface, captured more oxygen molecules, and produced more superoxide radicals and other types of ROS through dismutation or Fenton reaction^21^. Meanwhile, in the culture medium, COD-EBD had a lower absolute zeta potential than COD-BD (10.9 *vs* 12.6), and COD-CS had lower absolute zeta potential than COD-FL (5.75 *vs* 6.01). The crystals with low absolute value of zeta potential (COD-EBD and COD-CS) could enhance cell–crystal interactions^22^.

The COD-EBD and COD-BD crystals possess a tetragonal bipyramid structure consisting of four symmetry-related (100) faces and eight (101) faces constituting the two vertices. Previous research has shown that the surface concentrations of Ca^2+^ ions are obviously higher in the (100) face (0.0439 Ca^2+^/A^2^) than in the (101) face (0.0225 Ca^2+^/A^2^) of COD^23^. In addition, the adhesion force of the (100) face is higher than that of the (101) face. Atomic force microscopy (AFM) also showed that the adhesion force between the (100) face of COD crystals and AFM tips modified with S(CH_2_)_10_COO^−^ or S(CH_2_)_2_NHC(NH_2_^+^)NH_2_ is obviously higher than that of the (101) face. Therefore, the (100) face of COD crystals preferentially adheres to the plasma membrane of renal cells^24^. The COD-EBD with a large Ca^2+^ ion-rich (100) face theoretically promotes ROS generation and exacerbates cell injury. Meanwhile, the COD-CS crystals had sharply angled edges and tips, which seriously damaged the cell membrane, thereby upregulating membrane-associated NADPH oxidase and stimulating O_2_^−^ generation via NADPH oxidase^25^. Therefore, the ROS generation in the COD-CS-treated group was higher than that in the COD-FL-treated group.

Excessive ROS formation could overwhelm the natural scavenging activity of cells, which could induce mitochondrial membrane permeability, ultrastructural mitochondrial damage, mitochondrial depolarization, and respiratory chain disturbance^26^. Apoptosis and necrosis are often preceded by mitochondrial dysfunction accompanied with Δψm loss. Although the micron-sized COD crystals may not be in direct contact with intracellular organelles, all types of COD crystals decreased Δψm (Fig. 6). The crystals with large (100) faces (COD-EBD) and sharp edges (COD-CS) significantly decreased Δψm, which was consistent with the change in intracellular ROS generation. Cells undergo apoptosis or necrosis when the mitochondrial damage is severe, and the generated ROS overwhelm the natural scavenging activity of the cells^27^.

Cell death is a complicated pathological process that may be related to cell types, treatment time, crystal concentration, and even crystal physicochemical properties^28^. In the present study, Annexin V/PI staining results showed that all types of COD crystals induced different degrees of cell apoptosis and necrosis. The cell mortality in the COD-EBD-treated group was significantly higher than that in the COD-BD-treated group, which may be the reason why COD-EBD possessed the maximum (100) active crystal face. Another possible explanation for this behavior could be curvature differences between the COD-EBD and COD-BD crystals. The curvature differences of crystals could affect endocytosis and even the degree of membrane damage. The COD-EBD crystals display a larger contact area with the cell membrane than the COD-BD crystals because the longitudinal axis of the bipyramid interacts with the cell membrane. Therefore, COD-EBD caused more serious damage in HK-2 cells than COD-BD. The cell death induced by COD-EBD and COD-BD was mainly caused by apoptosis. The COD-CS and COD-FL crystals with small (100) faces but sharp edges decreased the cell death rates but increased the necrotic ratio in total mortality. Sharp crystals easily cause membrane damage, which leads to osmotic pressure imbalance across the membrane, thereby increasing necrotic rate^17^.

### Possible stone risk variation caused by COD crystals in various shapes

Aside from the effects of shape on crystal toxicity, the possible stone risk caused by various shapes of crystals flowing through the renal tubular lumen should also be different. In general, urinary flow through the renal tubules is laminar, in which case the flow velocity should be very small near the epithelium. Thus, these crystals located near the epithelial surface travel at a much slower speed, thereby extending the contact time between crystals near the tube wall and epithelial cells^29^. Crystal movement can be influenced by their morphology because of the Stokes’ drag. CaOx crystals with different morphologies display different drag magnitudes^30^. The area ratios and projection areas of crystals flowing through the renal tubular lumen are expected to dominate drag corrections^31^. The specific surface areas of COD-EBD and COD-CS crystals are larger than those of COD-BD and COD-FL crystals (Table 1). Crystals with large specific surface areas should possess higher Stokes’ drag and slower speed when they flow through the renal tubules. Slow flow increases the contact time between crystals and renal tubules; thus, the adhesion amounts of COD-EBD and COD-CS were larger than those of COD-BD and COD-FL (Fig. 8). Oxidative damage aggravates crystal adhesion and increases stone risk.

Among the crystals, the COD-EBD crystals exposed the largest active (100) face, which obviously increased the contact area between the crystals and cell surface. The adhesion force between COD-EBD and epithelial cells should be the highest among the four types of crystals because the adhesion force between the (100) face and cells is about two to three times than that of the (101) face^23^. Meanwhile, the COD-EBD crystals also caused the most serious injury to HK-2 cells. Thus, the crystals with large (100) faces theoretically have the highest risk of inducing stone formation. Champion *et al*. observed that oblong-shaped polystyrene-based microparticles exhibit higher attachment to the surface of macrophages compared with their spherical counterparts^32, 33^. Meanwhile, the COD-CS crystals with sharp edges flowing through the renal tubules easily scratch the cell membrane and induce inflammatory response. The membranous debris from the injured cell membrane promotes crystal nucleation and aggregation. Therefore, crystals with sharp edges cause great damage to the kidney and increase stone risk than round crystals.

## Conclusion

In this study, COD crystals with different shapes were utilized as model systems to investigate the influence of crystal shape on their cytotoxicity. The crystals with large (100) active crystal faces exhibited high cytotoxicity and possessed large adhesion area with the cell surface. These crystals also displayed a large adhesion amount on the cell surface. The crystals with sharp edges caused physical damage to the renal epithelial cells more easily than the round crystals. They also induced excessive ROS generation and caused injury to intracellular organelles. The results of this study suggest that the shape of crystals affects their cytotoxicity and increases potential stone risk. This study could serve as a theoretical basis for elucidating the mechanism of cell injury caused by CaOx crystals in various shapes and determining the causes of stone formation based on stone shape.

## Materials and Methods

### Reagents and apparatus

#### Materials

Human kidney proximal tubular epithelial (HK-2) cells were purchased from Shanghai Cell Bank, Chinese Academy of Sciences (Shanghai, China). Dulbecco’s modified Eagle’s medium (DMEM) and Fetal bovine serum were purchased from HyClone Biochemical Products Co., Ltd. (UT, USA). Penicillin and streptomycin were purchased from Beijing Pubo Biotechnology Co., Ltd. (Beijing, China). Cell Counting Kit-8 (CCK-8) was purchased from Dojindo Laboratories (Kumamoto, Japan). Lactate dehydrogenase (LDH) kit, hematoxylin–eosin (H&E) dye, 2′,7′-dichlorofluorescein diacetate dye (DCFH-DA) and 5,5′,6,6′-tetrachloro-1,1′,3,3′-tetraethyl-imidacarbocyanine iodide (JC-1), Annexin V-FITC, Propidium iodide (PI), and Rabbit anti-mouse IgG conjugated with fluorescein isothiocyanate (FITC-IgG) were all purchased from Shanghai Beyotime Bio-Tech Co., Ltd. (Shanghai, China). Cell culture plates were purchased from Wuxi Nest Bio-Tech Co., Ltd. (Wuxi, China). Calcium chloride (CaCl_2_), sodium oxalate (Na_2_Ox), ethylenediaminetetraacetic acid (Na_2_EDTA), dodecyl sodium sulfate (SDS) and the other conventional reagents were all analytically pure and purchased from Guangzhou Chemical Reagent Factory of China (Guangzhou, China).

#### Apparatus

The apparatus included X-L type environmental scanning electron microscope (SEM, Philips, Eindhoven, Netherlands), Nano-ZS nano particle sizer (Malvem, UK), D/max2400X X-ray powder diffractometer (Rigaku, Japan), tristar 3000 surface area and porosity analyzer (Micromeritics, American), enzyme mark instrument (Safire2™, Tecan, Männedorf, Switzerland) and fluorescence microscope (IX51, Olympus, Japan), and flow cytometer (FACS Aria, BD Corporation, Franklin Lakes, NJ, USA).

### Experimental methods

#### Preparation of COD crystals with various shapes

According to our previous study^34^, four shapes of COD crystals of approximately 5 μm in size were prepared by changing reactant concentration, reaction temperature, stirring speed, and additive. The four COD crystals with different shapes included cross-shaped (COD-CS), flower-like (COD-FL), bipyramid (COD-BD), and elongated–bipyramid (COD-EBD). The morphology and structure properties of prepared COD crystals were characterized with an X-L type environmental scanning electron microscope (SEM), an X-ray powder diffractometer, a Zetasizer Nano ZS90 apparatus, and a tristar 3000 surface area and porosity analyzer.

#### Cell culture and exposure to COD crystals of various shapes

Human kidney proximal tubular epithelial (HK-2) cells were cultured in a DMEM culture medium containing 10% fetal bovine serum, 100 U/mL penicillin-100 μg/mL streptomycin antibiotics with pH 7.4 at 37 °C in a 5% CO_2_ humidified environment. Upon reaching 80–90% confluent monolayer, cells were blown gently after trypsin digestion to form cell suspension for the following cell experiment. For experiments, the cells were seeded in culture plates at a density of 1 × 10^5^ cells/mL and allowed to attach for 24 h, then treated with varying shapes of COD crystals suspended in DMEM for a certain time. Cells maintained in DMEM without COD crystals were used as control group.

#### Cell viability assay

The cytotoxicity of varying shapes of COD crystals was evaluated by CCK-8 viability assay. HK-2 cells were exposed to 200, 400, 800 μg/mL COD-FL, COD-CS, COD-BD, and COD-EBD crystals for 6 h. Then 10 μL CCK-8 was added to each well and incubated for 2 h at 37 °C. Absorbance was measured by using the microplate reader at 450 nm.

1 $${\rm{C}}{\rm{e}}{\rm{l}}{\rm{l}}\,\mathrm{viability}( \% )\,=\frac{A\,({\rm{treatment}}\,{\rm{g}}{\rm{r}}{\rm{o}}{\rm{u}}{\rm{p}})-A\,({\rm{b}}{\rm{l}}{\rm{a}}{\rm{n}}{\rm{k}}\,{\rm{g}}{\rm{r}}{\rm{o}}{\rm{u}}{\rm{p}})}{A\,({\rm{c}}{\rm{o}}{\rm{n}}{\rm{t}}{\rm{r}}{\rm{o}}{\rm{l}}\,{\rm{g}}{\rm{r}}{\rm{o}}{\rm{u}}{\rm{p}})-A\,({\rm{b}}{\rm{l}}{\rm{a}}{\rm{n}}{\rm{k}}\,{\rm{g}}{\rm{r}}{\rm{o}}{\rm{u}}{\rm{p}})}\times 100$$

#### Hematoxyline-eosin (HE) staining

HE staining assay was performed on cells inoculated to 400 μg/mL COD-FL, COD-CS, COD-BD, and COD-EBD crystals. After 6 h incubation, the supernatant was removed by suction and washed three times with PBS. Afterwards, the cells were fixed with 4% paraformaldehyde for 15 min at room temperature. Cells were washed thrice with PBS. After fixation, the cells were stained with hematoxylin stain and incubated for 15 min. Then cells were washed with distilled water for 2 min to remove excess stain. After that, the cells were stained with eosin staining solution for 5 min. The cells were washed with distilled water for 2 min to remove excess eosin. After treatment, the cells were observed under the microscope.

#### Lactate dehydrogenase (LDH) release assay

The experimental model was divided into four groups: (A) cell–free culture medium wells (control wells of background); (B) control wells without drug treatment (sample control wells); (C) cells without drug treatment for the subsequent cleavage of the wells (sample maximum enzyme activity control wells); and (D) treated group with COD-FL, COD-CS, COD-BD, and COD-EBD crystals at the concentration of 200, 400, 800 μg/mL for 6 h (drug–treated wells). After incubation, the absorbance was analyzed at 490 nm with a reference wavelength of 620 nm according to the LDH kit instruction.

2 $$\mathrm{LDH} \% =\frac{A({\rm{Group}}\,{\rm{D}})-A({\rm{Group}}\,{\rm{A}})}{A({\rm{Group}}\,{\rm{C}})-A({\rm{Group}}\,{\rm{A}})}\times 100$$

#### Intracellular reactive oxygen species (ROS) assay

After the exposure of cells to 400 μg/mL COD-FL, COD-CS, COD-BD, and COD-EBD crystals for 6 h, the cells were suspended by pipetting, followed by centrifugation (1000 rpm, 5 min). The supernatant was aspirated and the cells were washed once with PBS and centrifuged again to obtain a cell pellet. The cells were resuspended by adding and thoroughly mixing 500 μL of PBS in a microcentrifuge tube. The samples were then stained with 2′, 7′-dichloro-fluorescein diacetate (DCFH-DA) for 20 min and analyzed by the flow cytometer.

#### Measurement of mitochondrial membrane potential (Δψm)

After the exposure of cells to 400 μg/mL COD-FL, COD-CS, COD-BD, and COD-EBD crystals for 6 h, the supernatant was aspirated and the cells were washed twice with PBS and digested with 0.25% trypsin. DMEM supplemented with 10% fetal bovine serum was then added to terminate digestion. The cells were suspended by pipetting, followed by centrifugation (1000 rpm, 5 min). The supernatant was aspirated and the cells were washed with PBS and centrifuged again to obtain a cell pellet. The cells were resuspended by adding and thoroughly mixing 200 μl of PBS in a microcentrifuge tube. Finally, the samples were stained with 5,5′,6,6′-tetrachloro-1,1′,3,3′-tetraethyl -imidacarbocyanine iodide (JC-1) and then analyzed by the flow cytometer.

#### Cell apoptosis and necrosis detection

Apoptosis and necrosis induced by varying shapes of COD crystals in HK-2 cells was measured by flow cytometer with Annexin V-FITC/PI double staining assay. Briefly, the cells were harvested after 12 h of exposure to 400 μg/mL COD-FL, COD-CS, COD-BD, and COD-EBD crystals, and then stained using Annexin V-FITC/PI cell death assay kit according to the manufacturer’s instructions. About 1.5 × 10^5^ cells were collected and washed with PBS (centrifuged at 1000 rpm for 5 min). The cells were resuspended in 200 μL binding buffer. Afterward, 5 μL Annexin V-FITC was added and then incubated in darkness at room temperature for 10 min. The cells were again resuspended in 200 μL binding buffer and stained with 5 μL PI. The prepared cells were then analyzed using a flow cytometer.

#### Quantitative analysis of adherent COD crystals by flow cytometry

According to our previous study^35^, FITC-IgG fluorescence-labeled COD crystals were prepared. Briefly, 10 μL of rabbit anti-mouse IgG conjugated with FITC (1 mg/mL) was mixed with 4 mL of COD crystals (800 μg/mL). The mixture was incubated at 37 °C overnight in the dark. Free FITC-IgG was removed using a dialysis bag (Mw 8000–14,000, Sigma, USA) for 48 h. Fluorescence-labeled COD crystals were harvested, washed, and dried.

The adhesion amount of FITC-IgG fluorescence-labeled COD crystals of various shapes on HK-2 cells was measured by flow cytometer. The experimental model was divided into two groups: (1) control group: in which only nomal serum-free culture medium was added; (2) adhesion group: cells were exposed to 400 μg/mL COD-FL, COD-CS, COD-BD, and COD-EBD crystals for 1 h. Afterward, the culture medium was removed by suction and the cells were washed twice with PBS (to eliminated the unbound crystals) followed by trypsinization, respectively. The cells were resuspended with 200 μL of PBS. The cellular adhesion of crystals was then quantitatively determined by flow cytometer, cells with positive FITC signal were directly counted as those with adherent crystals.

#### Statistical analysis

Statistical analyses were performed using the SPSS 13.0 software. Data were expressed as mean ± SD. Multiple group comparisons were performed using one-way ANOVA, followed by the Tukey post hoc test. If *p* < 0.05, there was significant difference; if *p* < 0.01, the difference was extremely significant; if *p* > 0.05, there was no significant difference.
